# Contribution of dental prostheses on the association between dental visits and self- reported chewing ability among older Japanese adults: a cross-sectional study

**DOI:** 10.1186/s12903-025-06673-0

**Published:** 2025-08-23

**Authors:** Mei Harada, Sachi Umemori, Yusuke Matsuyama, Hiroshi Nitta, Jun Aida

**Affiliations:** 1 Department of General Dentistry, Graduate School of Medical and Dental Sciences, Institute of Science Tokyo, Tokyo, Japan; 2 Department of Oral Health Sciences, School of Health Sciences, Meikai University, Japan 1 Akemi, Urayasu, Chiba 279-8550 Japan; 3 Department of Dental Public Health, Graduate School of Medical and Dental Sciences, Institute of Science Tokyo, Tokyo, Japan

**Keywords:** Dental prostheses, Mastication, Oral health, Universal health care, Mediation analysis, Cross-sectional studies

## Abstract

**Background:**

Dental treatment restores chewing ability among those with fewer teeth. However, few studies have evaluated the impact of dental visits on chewing ability. This study examined the mediating effect of dental prosthetic treatment for missing teeth on the association between dental visits and self-reported chewing ability in Japan, where national health insurance covers most of the basic prosthetic treatments.

**Methods:**

This cross-sectional study used data from the Japan Gerontological Evaluation Study. Self-reported questionnaires were distributed to older adults aged ≥ 65 years in 2022. The present analysis included those who answered the dental visit questionnaire and had ≤ 19 remaining teeth. The presence or absence of dental visits within one year was used as an explanatory variable, while the dental prosthetic treatments for missing teeth and the self-reported chewing ability were used as mediator and outcome, respectively. A causal mediation analysis was applied with possible confounders, including socioeconomic status.

**Results:**

Among 8,441 respondents, good chewing ability was reported by 56.3% of those who visited the dentist and 49.9% of those who did not visit the dentist within the past year. On mediation analysis, the dental prosthetic treatments accounted for 59.0% of the association between dental visits and good chewing ability (natural indirect effect, odds ratio = 1.25 [95% confidence interval: 1.18–1.33]).

**Conclusions:**

Although this cross-sectional study could not determine causality, reasonable association, dental visits were associated with better chewing ability through the dental prosthetic treatments for missing teeth, was suggested.

**Supplementary Information:**

The online version contains supplementary material available at 10.1186/s12903-025-06673-0.

## Introduction

Tooth loss and subsequent decline in chewing ability is potentially a significant global health burden among the older adults [1]. Impaired chewing ability has been associated with reduced nutritional intake [2, 3],diminished performance in activities of daily living (ADL) [4], decline in cognitive ability [5–7], and increased mortality risk [8–11]. According to the Global Burden of Disease Study, while the age-standardized prevalence of complete tooth loss, edentulism, decreased from 1990 to 2017, the number of edentulous people increased by 75.5% during this period, largely due to rapid population aging [12].

Previous research has reported that dental visits contribute to restoring chewing ability [13]. Mechanisms such as prevention of tooth loss through improvements in oral hygiene, prevention of caries and periodontal disease, and restoring chewing ability through dental prosthetic treatments have been suggested.

Dental prosthetic treatment is recognized for its ability to restore chewing ability [14–17], suggesting that for older individuals with significant tooth loss, restoration of chewing ability through dental prosthetic treatment is considered a crucial mechanism explaining the association between dental visits and restoring chewing ability.

However, social inequalities in access to dental care and dental prosthetic treatments for missing teeth have been reported in various countries [18–21]. Dental out-of-pocket costs vary by country due to differences in insurance coverage systems, with previous research suggesting that countries with lower dental out-of-pocket costs tend to have higher average dental visit frequencies [22]. Achieving Universal Health Coverage (UHC) is a key component of sustainable development goals aimed at reducing health disparities [22, 23], with recent emphasis on the necessity of including dental healthcare [1, 23, 24]. Japan's national health insurance system is globally recognized as one of the leading healthcare insurance systems, covering a wide range of dental prosthetic treatments and facilitating access to cost-effective dental prosthetic care. Among OECD countries, Japan achieves the lowest out-of-pocket rates and the highest utilization of dental healthcare services [22]. Therefore, dental prosthetic treatments are considered to make a significant contribution to restoring chewing ability in older individuals in Japan who have lost teeth.

Most previous studies evaluated chewing ability immediately after dental treatment, neglecting long-term functional outcomes as the prostheses deteriorate over time [25]. Thus, evaluating chewing ability among older adults after their last dental visit would address this critical gap. Therefore, this study aimed to evaluate whether dental prosthetic treatments mediate the association between dental visits and self-reported chewing ability among older adults who have experienced tooth loss.

## Methods

### Study population

**Fig. 1:**
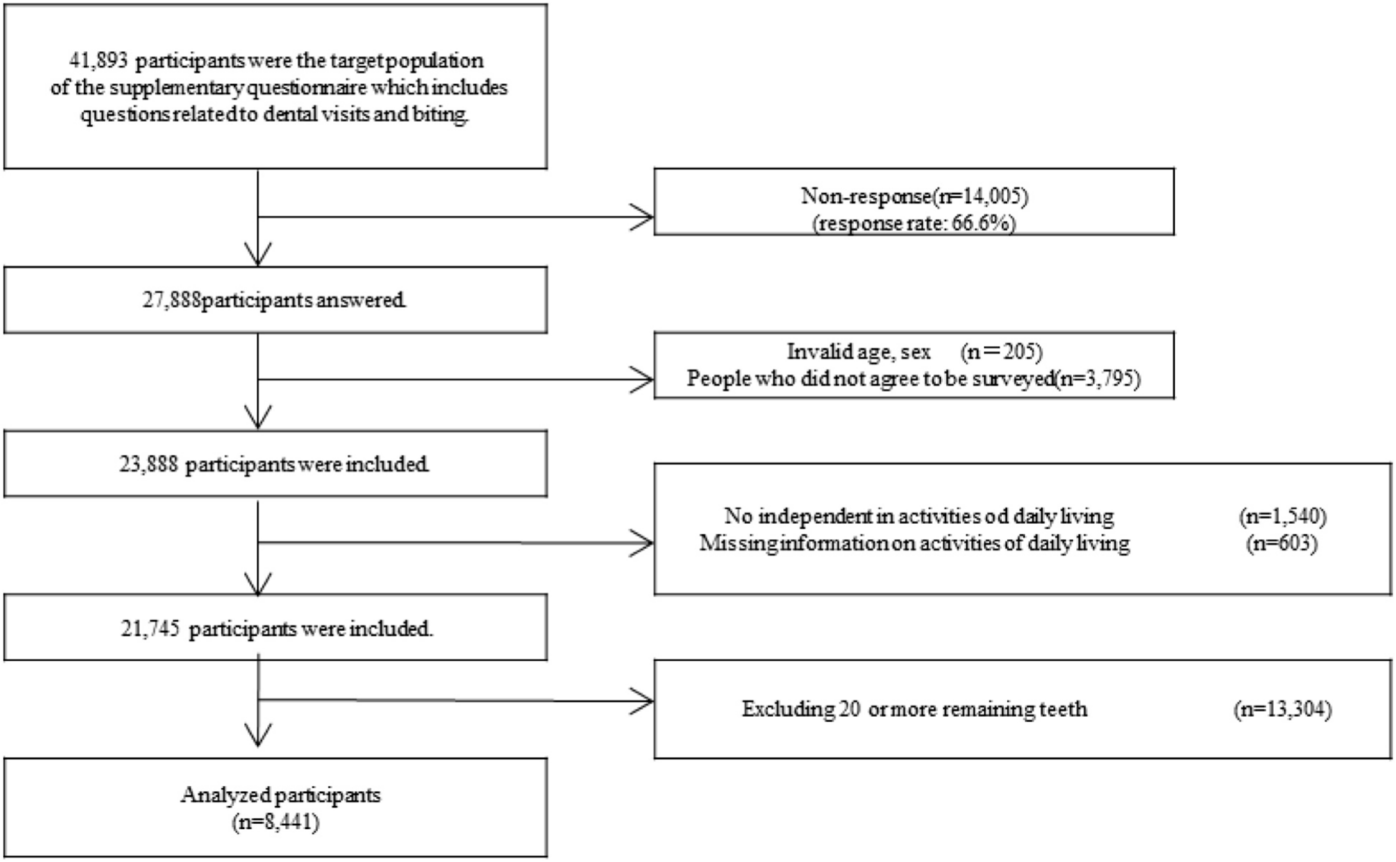
Flowchart of study participants

This cross-sectional study was based on self-reported questionnaire data from the 2022 survey of the Japan Gerontological Evaluation Study (JAGES). JAGES is an ongoing large-scale cohort study investigating health-related factors among individuals aged 65 years and older in Japan who do not receive public long-term care certification. The questionnaire was distributed by mail to 338,742 residents in 75 municipalities across Japan from December 2022 to January 2023, with responses collected by mail from consenting participants. The questionnaire consisted of a core survey and supplementary surveys in eight versions (A to H), and one version was randomly sent to one in eight of the participants. Version D included dental-related questions, and our study focused on respondents (*N* = 27,888) who completed this supplementary survey. Participants who reported limitations in basic ADL, such as walking, bathing, and toileting, although they did not strictly meet the eligibility criteria for public long-term care insurance benefits (*N*= 1,540), were excluded from the analysis. This exclusion was made because the JAGES survey targets independent older people who have not received long-term care certification [26]. However, since some individuals may not be officially certified but still lack functional independence, those with basic ADL limitations were also excluded to ensure a truly independent population and minimize potential confounding by underlying physical or cognitive impairments that may affect self-reported chewing ability.　Additionally, participants with 20 or more remaining teeth, indicating sufficient chewing ability [27, 28] were excluded. Finally, the analysis included 8,441 participants (average of the 10 imputed datasets). As this study is a secondary analysis of the 2022 JAGES, which is a nationwide survey conducted in collaboration between universities and municipal governments, the sample size was not based on a priori statistical power calculation but on the survey policies of municipal governments. The participants'flowchart is shown in Fig. 1. The questionnaire used in this study was developed by the Japan Gerontological Evaluation Study (JAGES), a nationwide survey. The questionnaire is available on the JAGES website after registration [29].

### Outcome variable

Self-reported chewing ability was used as the outcome variable. Responses to the self-reported questionnaire item,"Can you chew well?"were analyzed as the outcome variable. Participants who answered"yes"were categorized as having good chewing, while those who answered"no"were considered to have poor chewing. Chewing ability encompasses various physiological factors, including occlusal function, bite force, and jaw movements, which are critical components of masticatory performance. The question on chewing was intended to capture participants'subjective perception of their chewing function, reflecting their self-reported chewing ability. Self-reported chewing ability has been widely used in previous epidemiological studies and has shown acceptable reliability and validity as a proxy for objective masticatory performance [30].

### Explanatory variables

The presence or absence of dental visits was utilized as the explanatory variable. This was assessed using the question,"When did you last visit the dental clinic for'treatment'(including adjustment of dentures)?” The possible responses were:"during the past 6 months,""6 months to 1 year ago,""1 to 3 years ago,""more than 3 years ago,"and"I have never seen a dentist."In clinical settings, many dental treatments are completed within one year, after which maintenance becomes predominant. Therefore, for the primary analysis, participants were classified into two groups: “those with a dental visit within the past year” and “those with a dental visit more than one year ago or no history of dental visits. In addition to the primary analysis, two sensitivity analyses were conducted using different binary classifications. The first classification was “ever visited a dentist” vs. “never visited.” The second used a newly defined binary variable: “visited a dentist within the past 3 years” vs. “visited more than 3 years ago or never visited.” This latter classification was intended to further test the robustness of the observed associations by exploring an alternative threshold of dental visit recency.

### Mediators

Dental prosthetic treatment for missing teeth was employed as a mediating variable. Participants were asked,"Do you use removable dentures, dental bridges (fixed dentures), or have you had dental implants?"(Multiple answers were allowed.) Participants selected from options including"none,""removable dentures,""bridges,"and"dental implants."Those who selected any type of dental prosthesis were categorized as using dental prostheses, thereby dividing participants into two groups: “those using dental prostheses” and “those not using them.”

### Confounders

Based on previous studies and clinical knowledge, possible confounders were included [3, 11]. They included sex, age group (65–69 years, 70–74, 75–79, 80–84, 85 years or older), number of remaining teeth (0, 1–4, 5–9, 10–14, 15–19), equivalent income (< 2.00, 2.00–3.99, ≥ 4 million yen; JPY: 140 JPY ≈ 1 USD), education (≤ 9, 10–12, or ≥ 13 years), comorbidities (stroke, diabetes, cancer, and depression), instrumental activities of daily living (IADL), smoking status, and alcohol consumption. Depressive symptoms were assessed using the Geriatric Depressive Scale-15 with a cutoff of 5 points [31].

### Statistical analysis

**Fig. 2:**
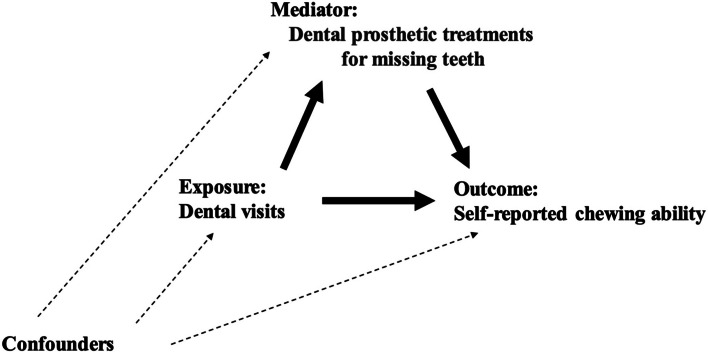
Directed acyclic graph of this study

A directed acyclic graph was used to illustrate the potential influences of dental visits on chewing ability, along with mediating and confounding factors (Fig. 2). At first, we fitted logistic regression models to estimate the odds ratios (ORs) and 95% confidence intervals (CI) for dental visit in relation to chewing ability. First, we examined the association between dental visits and self-reported chewing ability through univariable analysis (Model 1). Second, sex, age, number of remaining teeth, equivalent income, years of education, comorbidities (stroke, diabetes, cancer, depression), IADL, depressive symptoms, smoking status, and alcohol consumption were adjusted (Model 2). Finally, dental prosthetic treatment for missing teeth was added to Model 2 (Model 3).

Then, the causal mediation approach was implemented with parametric regression models to estimate the natural direct effect, natural indirect effect (NIE) (with dental prostheses as the mediator), and total effect by the “PARAMED” command in STATA software [32]. The direct effect indicates the impact of dental visits on chewing ability not through the dental prosthetic treatments for missing teeth, whereas the indirect effect reflects the impact of dental visits on chewing ability mediated by the dental prosthetic treatments for missing teeth. The sum of the direct and indirect effects is represented as the total effect. Both the outcome and mediator models utilized logistic regression models. The magnitude of the associations was estimated using ORs, and the proportion mediated was calculated by log-scale. In addition to the primary analysis, we conducted two types of sensitivity analyses using alternative binary classifications of dental visits. The first classification categorized participants as “ever visited a dentist” vs. “never visited.” The second used a newly defined binary variable: “visited a dentist within the past 3 years” vs. “visited more than 3 years ago or never visited.” Logistic regression and mediation analyses were performed for both classifications, and the proportion mediated was calculated. Since the primary analysis was based on data processed by multiple imputation using chained equations (MICE), we also conducted complete-case analyses to assess the robustness of the findings.

To reduce selection bias, missing data were imputed using multiple imputation by chained equations (MICE), constructing 10 imputed datasets [33]. Among 8,441 participants, data for 3,615 participants were imputed. Estimates from each imputed dataset were combined using Rubin's rules [34]. Statistical significance was set at α = 0.05 for all analyses. Statistical analyses were conducted using Stata/MP version 18.0 (Stata Corp., College Station, Texas, USA).

## Results

Table 1 summarizes participants’ characteristics after MICE. The average age was 76.5 years (SD = 6.5), 49.9% were female, and 41.2% had fewer than 10 remaining teeth. In total, 53.9% had visited a dentist within the past year, and 63.0% reported having good chewing ability. Furthermore, among those who had visited a dentist within the past year, 56.3% reported good chewing ability, whereas this proportion was 43.7% among those who had not.

**Table 1:** Descriptive characteristics of the participants after multiple imputation (*N* = 8,441)

	**Number of participants (%)**	**Participants received dental treatment within the past year (%)**	**Participants received no dental treatment in the past year (%)**
	8,441	53.9	46.1
Can you chew well?			
Yes	5,320 (63.0)	56.3	43.7
No	3,121 (37.0)	49.9	50.1
Dental prosthetic treatments for missing teeth			
Yes	7,414 (87.8)	57.4	42.6
No	1,027 (12.2)	28.8	71.2
Number of remaining teeth			
0 teeth	1,470 (17.4)	26.9	73.1
1–4 teeth	1,202 (14.2)	48.2	51.8
5–9 teeth	1,709 (20.2)	57.2	42.8
10–14 teeth	1,953 (23.1)	62.7	37.3
15–19 teeth	2,107 (25.0)	65.3	34.7
Sex			
Male	4,228 (50.1)	51.6	48.4
Female	4,213 (49.9)	56.3	43.7
Age			
65–69 years	1,276 (15.1)	51.3	48.7
70–74 years	2,260 (26.8)	54.1	45.9
75–79 years	2,089 (24.7)	57.3	42.7
80–84 years	1,721 (20.4)	55.8	44.2
≥ 85 years	1,095 (13.0)	47.3	52.7
Equivalent income (100JPY = 1USD)			
< 200 million JPY	4,940 (58.5)	50.8	49.2
200–399 million JPY	2,731 (32.4)	58.1	41.9
≥ 400 million JPY	769 (9.1)	59.1	40.9
Education			
≤ 9 years	2,561 (30.3)	48.3	51.7
10–12 years	3,667 (43.4)	55.8	44.2
≥ 13 years	2,213 (26.2)	57.4	42.6
Instrumental activities of daily living (IADL)^a^	1.8 ± 0.02	1.7 ± 0.03	2.1 ± 0.04
Depressive symptoms (GDS)			
Non	6,150 (72.9)	55.8	44.2
Mild	1,803 (21.4)	49.5	50.5
Severe	488 (5.8)	46.8	53.2
History of depression			
No	8,363 (99.1)	53.9	46.1
Yes	78 (0.9)	56.6	43.4
History of cancer			
No	8,126 (96.3)	53.8	46.2
Yes	315 (3.7)	57.8	42.2
History of diabetes			
No	7,010 (83.0)	54.4	45.6
Yes	1,431 (17.0)	51.8	48.2
History of stroke			
No	8,217 (97.3)	53.9	46.1
Yes	224 (2.7)	56.4	43.6
Smoking status			
Current	1,177 (13.9)	44.9	55.1
Past	2,784 (33.0)	53.8	46.2
Never	4,480 (53.1)	56.3	43.7
Drinking habits			
Current	3,192 (37.8)	55.3	44.7
Past	1,181 (13.9)	48.5	51.5
Never	4,068 (48.4)	54.4	45.6

^a^Average of continuous values from 0 ~ 13 (standard deviation)

Table 2 presents the results of logistic regression analyses conducted after multiple imputations. After imputation and adjustment for all confounders, a positive association between dental visits within the past year and good chewing ability was observed (Model 2: OR = 1.35; 95% CI: 1.22–1.49). Similarly, Model 3, which adjusted for the dental prosthetic treatments for missing teeth, revealed that the association between dental visits and chewing ability was attenuated (Model 3: OR = 1.17; 95% CI: 1.06–1.30).

**Table 2:** Odds ratio of dental visits for good chewing ability (MI, logistic regression) (*N* = 8,441)

	**After applying multiple imputation**
	**OR (95%CI)**
Model 1	1.29* (1.18–1.42)
Model 2	1.35* (1.22–1.49)
Model 3	1.17* (1.06–1.30)

Model 1: Univariable logistic regression

Model 2: Model 1 + sex, age, income, education, instrumental activities of daily living (IADL), depressive symptoms, comorbidities (cancer, diabetes, depression, and stroke), smoking status, drinking habits, number of remaining teeth adjusted

Model 3: Model 2 + dental prosthetic treatments for missing teeth

*CI* Confidence interval, *OR* Odds ratio

^*^P < 0.01

To explore the mechanism underlying the association between dental visits and chewing ability, we conducted a causal mediation analysis to determine whether this association was mediated by dental prosthetic treatment for missing teeth. In this framework, the total effect (TE) of dental visits on chewing ability is decomposed into a natural direct effect (NDE) and a natural indirect effect (NIE). The NDE represents the portion of the effect that occurs through pathways other than prosthetic treatment, while the NIE captures the portion mediated through the prosthetic intervention. The analysis revealed a significant mediation effect (Table 3), with a NIE of OR: 1.25; 95% CI: 1.18–1.33]). Dental prosthetic treatment mediated 59.0% of the total effect of dental visits on chewing ability. This finding suggests that the improvement in chewing ability following dental visits is largely attributable to receiving prosthetic treatment for missing teeth. These results underscore the key role of prosthetic intervention as the main pathway linking dental visits to better oral function.

**Table 3:** Mediating effect of prosthetic treatment on dental visits and chewing ability (MI) (*N* = 8,441)

	**OR**	**(95% CI)**
Total effect	1.46	(1.31, 1.63)
NDE	1.21	(0.92, 1.58)
NIE	1.25	(1.18, 1.33)
Proportion mediated	59.0%	

*CI* Confidence interval, *NDE* Natural direct effect, *NIE* Natural indirect effect, *OR* Odds ratio

Additionally, we conducted a complete-case analysis using the same definition as the primary analysis (“within the past year” vs. “more than 1 year ago or never visited”) to assess the robustness prior to multiple imputation. Logistic regression results were consistent with those of the primary analysis, showing a positive association between dental visits and good chewing ability (Model 1: OR = 1.27, 95% CI: 1.13–1.43; Model 2: OR = 1.33, 95% CI: 1.17–1.51; Model 3: OR = 1.14, 95% CI: 1.00–1.30). The total effect was OR 1.49 (95% CI: 1.29–1.73), with NIE of 1.32 and NDE of 1.14, resulting in a proportion mediated of 69.6%. These findings were consistent with those of the main analysis.

## Discussion

Most previous studies evaluated chewing ability immediately after dental treatment. Over time, there is a possibility that the function of the prostheses may deteriorate. This study estimated how dental visits and the use of prostheses contribute to chewing ability among older people with varying lengths of time since the prosthetic treatment. From this study, it is suggested that the dental visits within the past year among older individuals with fewer than 19 remaining teeth were associated with chewing ability recovery through dental prosthetic treatments for missing teeth. Dental prostheses explained approximately 59.0% of the association.

In sensitivity analyses using alternative categorizations of dental visits, we observed similar associations. When we categorized participants based on the presence or absence of any dental visit, approximately 61.6% of the association was mediated. Additionally, using a different sensitivity analysis with the classification of “visited a dentist within the past 3 years” vs. “visited more than 3 years ago or never visited,” 49.1% of the total effect was mediated through prosthetic treatment. These results imply that dental prostheses influence chewing ability following tooth loss.

Previous studies conducted in various countries have consistently reported that dental prostheses, such as dentures, dental bridges, and implants, can recover chewing efficiency following tooth loss [16, 17, 25, 35, 36]. Liang et al. conducted a systematic review on the impact of removable partial dentures (RPDs) in subjects with moderate to severe shortened dental arches, demonstrating that RPDs can restore approximately 50% of chewing ability, with better performance as the number of artificial teeth in the RPD increases [25]. Oki et al. studied subjects classified as Kennedy Class II (partially edentulous with unilateral free-end saddles) who underwent dental prosthetic treatments using either RPDs or implant-supported fixed prostheses and reported improvements in chewing ability before and after treatment in all examined cases [37]. Our study design was different from these studies; it used simple measurements of prosthetic treatment and self-reported chewing ability. Also, the present study adjusted various confounding factors and had a large sample size. The results of the present study are consistent with previous findings.

Furthermore, regarding the association between dental visits and chewing ability, it has been reported that individuals who do not receive regular dental care tend to experience deterioration in oral health [38, 39]. In previous studies conducted in Japan, Fujii and Kikui reported that regular dental utilization affects objectively measured chewing ability [13, 40]. However, these studies did not adjust for an important social factor, socioeconomic status (SES). Higher SES is associated with better health behaviors, such as dental visits [41], and better chewing ability by reducing the risk of tooth loss [28, 42]. Therefore, considering SES is crucial for addressing confounding bias.

From a clinical perspective, our findings indicate that dental prosthetics treatment in older individuals with poor chewing ability due to tooth loss can significantly enhance masticatory ability. From a public health perspective, previous research has demonstrated social inequalities in dental visits and denture use [43–47]. Therefore, dental treatments need to be incorporated into universal health coverage so that everyone can receive dental care, including prosthetic treatment, at an acceptable price [48].

This study has several limitations. Firstly, it relies on self-reported questionnaire data, which may introduce inaccuracies. Additionally, as all variables in this study (dental visits, prosthesis use, and chewing ability) were self-reported, potential recall and reporting biases may have influenced the findings. However, previous research has shown that there is a significant correlation between clinically measured and self-reported chewing status, as well as consistency between clinically assessed remaining teeth and self-reported tooth counts [49]. Moreover, the question on chewing ability in this study is similar to measures used in various oral health-related quality of life scales [50]. A detailed assessment of dental status, such as occlusal units, was not possible. Nevertheless, chewing ability is a complex function influenced by multiple physiological factors, such as the occlusal contact area formed by teeth, bite force generated by masticatory muscles, neuromuscular coordination of chewing movements, and sufficient saliva production. Sensory factors, including food texture and taste, further impact chewing efficiency [51]. As this study defined chewing ability based on the participants'perceived responses, objective measures, such as masticatory performance using fragmentation tests, were not captured. These limitations highlight the need for future studies incorporating both subjective and objective assessments to comprehensively evaluate chewing function.

Future research utilizing clinical investigations is essential for obtaining more accurate measurements. Second, this study used a cross-sectional design, and hence cannot infer the temporal association between dental visit, dental prosthetic treatments for missing teeth, and chewing status. Despite using causal mediation analysis, the cross-sectional design precludes establishing causality between dental visits and chewing ability. Although we employed causal mediation analysis to explore possible pathways between dental visits and chewing ability, the cross-sectional design of this study precludes definitive conclusions about temporal relationships or causal inferences. Further cohort studies are required. Third, although this study applied multiple imputation to handle missing data, the large number of missing cases due to the older population still raises concerns about potential selection bias.

This study has several strengths. First, it utilized a relatively large sample size of 8,441 participants across Japan. This large sample size enhances the statistical power of the analysis. While the study included a diverse range of municipalities, they were not randomly selected. Therefore, although the findings may offer insights applicable to many older adults in Japan, the generalizability to the entire population may be limited. Second, this study provides insight into the universal health coverage of dental prosthetic treatments. In Japan, universal health insurance covers dental prosthetic treatments. Finally, this study shows the average state of chewing ability in older people with few teeth. Many previous studies have reported improvements in chewing ability immediately after treatment. However, there is a possibility that the function of the denture may deteriorate after treatment. Our study examined two time periods after the dental visit and showed that the contribution of the prostheses to chewing may still be present even after a long time has elapsed since the dental visit. While this study was conducted in Japan, where dental prosthetic treatments are widely accessible under universal health coverage, our findings may also be informative for other aging populations. However, the applicability of the results may vary depending on the cultural, economic, and healthcare contexts of different countries. Therefore, further research in diverse international settings is warranted to validate these associations.

## Conclusions

In this study, dental prosthetic treatment for missing teeth was suggested to play a crucial role in chewing ability among older adults with fewer than 19 remaining teeth. Integration of prosthetic treatment into universal health coverage is desired in other countries, and this need is expected to grow increasingly in the future.

## Supplementary Information

Supplementary Material 1.

## Data Availability

The datasets used and/or analyzed during the current study are available from the corresponding author on reasonable request.

## Abbreviations

ADL: Activities of Daily Living
UHC: Universal Health Coverage
JAGES: Japan Gerontological Evaluation Study
IADL: Instrumental Activities of Daily Living
ORs: Odds Ratios
CI: Confidence Intervals
NIE: Natural Indirect Effect
MICE: Multiple Imputation by Chained Equations
RPDs: Removable Partial Dentures
SES: Socioeconomic Status
