# Supplementary material for: Contribution of dental prostheses on the association between dental visits and self- reported chewing ability among older Japanese adults: a cross-sectional study
Source: BMC Oral Health. 2025 Aug 23;25:1363. doi: 10.1186/s12903-025-06673-0 (PMC12374398; doi:10.1186/s12903-025-06673-0)
Supplement: Supplementary file 1 — Supplementary Material 1. [file 12903_2025_6673_MOESM1_ESM.pdf]

Supplementary Table S1. Descriptive characteristics of the participants after multiple imputation for sensitivity analysis (N=8,434)

	Number of Participants (%)	Participants received dental treatment within the past year (%)	Participants received no dental treatment in the past year (%)
	**8,434**	**97.1**	**2.9**
**Can you chew well?**			
Yes	5,313 (63.0)	98.0	2.0
No	3,121 (37.0)	95.5	4.5
**Dental prosthetic treatment for missing teeth**			
Yes	7,409 (87.8)	98.6	1.4
No	1,025 (12.2)	85.8	14.2
**Number of remaining teeth**			
0 teeth	1,471 (17.4)	94.6	5.4
1–4 teeth	1,194 (14.2)	96.2	3.8
5–9 teeth	1,715 (20.3)	98.0	2.0
10–14 teeth	1,950 (23.1)	97.7	2.3
15–19 teeth	2,110 (25.0)	98.0	2.0
**Sex**			
Male	4,227 (50.1)	96.4	3.6
Female	4,207 (49.9)	97.8	2.2
**Age**			
65–69years	1,275 (15.1)	97.1	2.9
70–74years	2,261 (26.8)	96.8	3.2
75–79years	2,086 (24.7)	97.2	2.8
80–84years	1,719 (20.4)	97.6	2.4
≥85years	1,092 (12.9)	96.6	3.4
**Equivalent income (100JPY=1USD)**			
<200 million JPY	4,934 (58.5)	96.2	3.8
200–399 million JPY	2,730 (32.4)	98.2	1.8
≥400 million JPY	770 (9.1)	98.6	1.4

**Education**				
	≤9 years	2,556 (30.3)	95.9	4.1
	10–12 years	3,663 (43.4)	97.4	2.6
	≥13 years	2,214 (26.3)	97.9	2.1
**Instrumental activities of daily living (IADL)†**		1.8±0.02	1.8±0.02	2.3 ±0.16
**Depressive symptoms (GDS)**				
	Non	6,145 (72.9)	97.4	2.6
	Mild	1,803 (21.4)	95.9	4.1
	Severe	486 (5.8)	96,7	3.3
**History of depression**				
	No	8,356 (99.1)	97.1	2.9
	Yes	78 (0.9)	98.7	1.3
**History of cancer**				
	No	8,118 (96.3)	97.0	3.0
	Yes	316 (3.7)	98.5	1.5
**History of diabetes**				
	No	7,004 (83.0)	96.9	3.1
	Yes	1,430 (17.0)	97.8	2.2
**History of stroke**				
	No	8,210 (97.3)	97.1	2.9
	Yes	224 (2.7)	96.8	3.2
**Smoking status**				
	Current	1,177 (14.0)	95.8	4.2
	Past	2,783 (33.0)	97.6	2.4
	Never	4,474 (53.0)	97.1	2.9
**Drinking habits**				
	Current	3,193 (37.9)	97.3	2.7
	Past	1,178 (14.0)	96.9	3.1
	Never	4,064 (48.2)	96.9	3.1

†Average of continuous values from 0~13 (standard deviation)

Supplementary Table S2. Descriptive characteristics of the participants before multiple imputation (complete-case analysis) (N=4,826)

	Number of Participants (%)	Participants received dental treatment within the past year (%)	Participants received no dental treatment in the past year (%)
	**4,826**	**56.8**	**43.2**
**Can you chew well?**			
Yes	3,017 (62.5)	59.1	40.9
No	1,809 (37.5)	53.1	46.9
**Dental prosthetic treatment for missing teeth**			
Yes	4,269 (88.5)	60.2	39.8
No	557 (11.5)	31.1	68.9
**Number of remaining teeth**			
0 teeth	699 (14.5)	27.0	73.0
1–4 teeth	666 (13.8)	51.5	48.5
5–9 teeth	992 (20.6)	59.8	40.2
10–14 teeth	1,186 (24.6)	62.8	37.2
15–19 teeth	1,283 (26.6)	68.0	32.0
**Sex**			
Male	2,699 (55.9)	54.2	45.8
Female	2,127 (44.1)	60.2	39.8
**Age**			
65–69years	846 (17.5)	54.1	45.9
70–74years	1,455 (30.1)	56.0	44.0
75–79years	1,171 (24.3)	60.5	39.5
80–84years	863 (17.9)	59.1	40.9
≥85years	491 (10.2)	51.1	48.9
**Equivalent income (100JPY=1USD)**			

<200 million JPY	2,599 (53.9)	53.6	46.4
200–399 million JPY	1,736 (35.9)	60.5	39.5
≥400 million JPY	491 (10.2)	60.9	39.1
**Education**			
≤9 years	1,237 (25.6)	51.1	48.9
10–12years	2,192 (45.4)	58.5	41.5
≥13 years	1,397 (28.9)	59.3	40.7
**Instrumental activities of daily living (IADL)†**	1.8±0.03	1.6±0.03	2.0±0.04
**Depressive symptoms (GDS)**			
Non	3,574 (74.1)	58.6	41.4
Mild	994 (20.6)	51.4	48.6
Severe	258 (5.3)	52.7	47.3
**History of depression**			
No	4,780 (99.0)	56.8	43.2
Yes	46 (1.0)	56.5	43.5
**History of cancer**			
No	4,617 (95.7)	56.7	43.3
Yes	209 (4.3)	58.9	41.1
**History of diabetes**			
No	3,980 (82.5)	57.3	42.7
Yes	846 (17.5)	54.6	45.4
**History of stroke**			
No	4,703 (97.5)	56.9	43.1
Yes	123 (2.5)	56.1	43.9
**Smoking status**			
Current	753 (15.6)	46.7	53.3
Past	1,780 (36.9)	57.1	42.9
Never	2,293 (47.5)	60.0	40.0
**Drinking habits**			
Current	1,998 (41.4)	57.2	47.8
Past	695 (14.4)	52.1	47.9
Never	2,133 (44.2)	58.0	42.0

†Average of continuous values from 0~13 (standard deviation)

Supplementary Table S3. Odds ratio and 95% CI of sensitivity analysis for presence of absence of good chewing ability by logistic regression (MI, logistic regression) (N=8,434)

	After applying multiple imputation
	OR (95%CI)
**Model 1**	2.36*(1.77–3.15)
**Model 2**	2.39*(1.76–3.25)
**Model 3**	1.54*(1.12–2.11)

* :P<0.01; CI, confidence interval; OR, odds ratio

Model 1: Univariable logistic regression

Model 2: Model 1 ＋ sex, age, income, education, instrumental activities of daily living (IADL), depressive symptoms, comorbidities (cancer, diabetes, depression, and stroke), smoking status, drinking habits, number of remaining teeth adjusted

Model 3: Model 2 + dental prosthetic treatments for missing teeth

Supplementary Table S4. Sensitivity analysis of the mediating effect of dental prosthetic treatments for missing teeth in the association between dental visits and presence or absence of good chewing ability (MI) (N=8,434)

	**OR**	**(95% CI)**
Total effect	2.74	(1.89, 3.98)
NDE	1.47	(1.01, 2.15)
NIE	1.86	(1.26, 2.74)
Proportion mediated	61.6%	

CI, confidence interval; NDE, natural direct effect; NIE, natural indirect effect; OR, odds ratio

Supplementary Table S5. Odds ratio of dental visits for good chewing ability (complete-case analysis, logistic regression) (N=4,826)

	**Before applying multiple imputation**
	**OR (95%CI)**
**Model 1**	1.27**(1.13-1.43)
**Model 2**	1.33**(1.17-1.51)
**Model 3**	1.14*(1.00-1.30)

* :P<0.05 * :P<0.01; CI, confidence interval; OR, odds ratio

Model 1: Univariable logistic regression

Model 2: Model 1 ＋ sex, age, income, education, instrumental activities of daily living (IADL), depressive symptoms, comorbidities (cancer, diabetes, depression, and stroke), smoking status, drinking habits, number of remaining teeth adjusted

Model 3: Model 2 + dental prosthetic treatments for missing teeth

Supplementary Table S6. Mediating effect of prosthetic treatment on dental visits and chewing ability (complete-case analysis) (N=4,826)

	**OR**	**(95% CI)**
Total effect	1.49	(1.29, 1.73)
NDE	1.14	(1.00, 1.30)
NIE	1.32	(1.21, 1.43)

Proportion mediated	69.6%

CI, confidence interval; NDE, natural direct effect; NIE, natural indirect effect; OR, odds ratio
